# Exploration of the academic lives of students with disabilities at South African universities: Lecturers’ perspectives

**DOI:** 10.4102/ajod.v6i0.316

**Published:** 2017-03-30

**Authors:** Oliver Mutanga, Melanie Walker

**Affiliations:** 1 Institute of Health and Society, Faculty of Medicine, University of Oslo, Norway; 2 Centre for Research on Higher Education and Development, University of the Free State, South Africa

## Abstract

**Background:**

A decade has passed since South Africa signed and ratified the Convention on the Rights of Persons with Disabilities, a human rights treaty that protects the rights and dignity of people with disabilities. However, not much have changed for students with disabilities.

**Objectives:**

The aim of this study was to explore lecturers’ experiences with, and perspectives on, disability as well as with students with disabilities. It was hoped that this would contribute to the ongoing policy debates about diversity, inclusion and support for students with disabilities at universities.

**Methods:**

In an effort to understand the lives of students with disabilities better, a study which included students with disabilities, lecturers and disability supporting staff was conducted at two South African universities – University of the Free State and University of Venda. The paper takes a snapshot view of four lecturers and their perceptions of the lives of students with disabilities at their respective universities.

**Results and Conclusion:**

Although most disability literature report students with disabilities blaming lecturers for their failure to advance their needs, this paper highlights that the education system needs to be supportive to lecturers for the inclusive agenda to be realised. An argument is made for a more comprehensive approach towards a national disability policy in higher education involving many stakeholders. Without a broader understanding of disability, it will be difficult to engage with the complex ways in which inequalities emerge and are sustained.

## Introduction

The aim of this paper is to understand lecturers’ thoughts and views on how the needs of students with disabilities^1^ are acted upon at these selected universities. Insights from these lecturers provide data that are helpful in comprehending the experiences of students with disabilities in South African universities. This contributes to our understanding of lecturers’ roles in the lives of students with disabilities, the barriers they face and also the support they might need to enable them to deal with diversity in higher education.

As of 2016, there has been no legislation that specifically looks at disability issues in South African higher education. With specific reference to disability, and to facilitate the inclusion and participation of people with disabilities in all spheres of the economy, the National Commission on Special Education Needs and Training and the National Committee on Education Support Services were appointed in 1996. Their findings (DoE 1997), produced in 1997, stated that:

The primary challenge to higher education institutions at present is to actively seek to admit learners with disabilities who have historically been marginalised at this level, providing them with opportunities to receive the education and training required to enter a variety of job markets. Alongside this is the challenge to develop the institution’s capacity to address diverse needs and address barriers to learning and development. This includes not only learners with disabilities, but all learners. This requires that adequate enabling mechanisms be put in place to ensure that appropriate curriculum and institutional transformation occurs, and that additional support is provided where needed. (p. 126)

This report pointed out that there was a need to admit more students with disabilities and to facilitate their full participation (Matshedisho 2007). The Integrated National Disability Strategy (INDS) was introduced in 1997 with the intention to both guide and support increased employment of, and to some degree to serve, people with disabilities within government structures. Former President, Thabo Mbeki (Office of the Deputy President [ODP] 1997), acknowledged this:

This White Paper [*INDS*] represents the government’s thinking about what it can contribute to the development of disabled people and to the promotion and protection of their rights. We believe in a partnership with disabled people. Therefore, the furtherance of our joint objectives can only be met by the involvement of disabled people themselves. (p. 2)

The government thus recognised both the need for the rights of disabled people to be protected as well as their involvement and participation in matters affecting their lives (Howell 2005).

In 2001, the government released the National Plan for Higher Education (NPHE). The NPHE outlines the framework and mechanisms through which the policy goals and transformation imperatives of the White paper 3 and *Higher Education Act* could be implemented (Ministry of Education [MoE] 2001). Among other things, the NPHE established indicative targets for the size and shape of the higher education system. Although there is no reference to students with disabilities, of particular relevance in the context of this study is the strong focus on equity issues through the identification of non-traditional students as a target group for inclusion in higher education.^2^ It also recommended that participation rates in higher education should increase from 15% to 20% by 2016 (MoE 2001). In the same manner as the INDS, the MoE lamented a lack of data on the status of students with disabilities in South African higher education (MoE 2001). Again, in the same year, the Education White Paper 6 primarily covering the education of students with disabilities at the primary and secondary school level was released, stating that students with disabilities should have fair and equal opportunities to access and succeed in higher education.^3^ The paper provided guidelines to remove obstacles and challenges that hinder students with disabilities’ access and participation. It was also suggested that higher education institutions’ response to the needs of students with disabilities was important and regional collaboration among them was important in this regard. However, although it purports to cover inclusive education and participation of students with disabilities in higher education, some of its provisions seem to suggest otherwise. For instance, Section 2.2.5.3 (DoE 2001) states that:

It will not be possible to provide relatively expensive equipment and other resources, particularly for blind and deaf students, at all higher education institutions. Such facilities will therefore have to be organised on a regional basis. (p. 31)

There are no details on how this can be implemented in practice. Moreover, there are no legal sanctions for failure to comply with this duty. By insisting that it ‘will not be possible’ to provide equipment and resources to a section of the population, justifying this in economic terms, the paper arguably risks perpetuating inequalities. Instead of the assurance of service provision, this paper places the burden on disabled students to justify their right to be included in higher education in such a way that does not place economic burdens on higher education institutions.

In 2013, the White paper for Post-School Education and Training was released. It states that higher education institutions need to accommodate students with diverse needs and remove barriers that hinder the development of all students. This is a positive move towards inclusive practices in higher education. The paper states that the government remains committed to improving access and success for ‘non-traditional students’ (disabled, black and female students). Therefore, it prioritises increasing student participation rates and improving their performance, success and throughput rates. The paper (Department of Higher Education and Training [DHET] 2013) further says that it will develop a strategic policy framework to drive this initiative:

The DHET will develop a strategic policy framework to guide the improvement of access to and success in post-school education and training for people with disabilities. The framework will require all post-school institutions to address policy within institutional contexts and to develop targeted institutional plans to address disability. (p. xv)

This policy framework is problematic in that it fails to recognise heterogeneity within the persons with disabilities and lumps all ‘people with disabilities’ into one group. A one-size-fits-all approach has the danger of failing to meet the needs of individuals with certain impairments. In December 2014, a Ministerial Committee was set up by the Minister of Higher Education and Training to develop the strategic policy framework as articulated in the 2013 White paper. The committee is still working on that framework. Even though certain elements require ongoing critical debate, inclusive initiatives in South African higher education as explicated in various policy documents are currently being pushed and action is evident. Notwithstanding these significant policy initiatives, a number of challenges continue to confront higher education, including universities. For example, the responsibility of ensuring disability rights in higher education is relegated only to one department – DHET. Furthermore, some goals and values are in tension with one another; for example, pursuing social equity and redress alongside the production of high-quality graduates in the context of inadequate public funding and initiatives to support underprepared students (who include students with disabilities). While the policies are impressive on paper, the real question is why there are still challenges within the South African higher education system. Commenting on the issues of inclusion, Carrim (2002) argues that:

Although it would be fair to state that South African education and training legislation and policies promote an expanded and rich use of the notion of inclusion, it cannot be assumed that this is reflective of current, and emerging, practices. Instead, mounting evidence seems to suggest that various forms of exclusion still prevail throughout the system currently. (p. 14)

This calls for more careful consideration of the equity issues and the barriers within universities which restricts full inclusion and participation of students with disabilities. The current policy momentum clears the way for a platform to contribute the findings from this study.

### Students with disabilities’ perceptions of their lecturers

Few studies have investigated the experiences and perspectives of lecturers regarding the experiences of students with disabilities at South African universities. Among these studies, a degree of scepticism about disability among able-bodied lecturers was identified, including concerns about the fairness of allowing students with disabilities greater access to materials and additional contact with staff and questions about whether some students with disabilities should be given university places at all (Mayat & Amosun 2011; Riddell et al. 2007). Most South African disability studies (Engelbrecht & de Beer 2014; Ntombela & Soobrayen 2013; Ohajunwa et al. 2014; Swart & Greyling 2011; Tugli et al. 2013) have explored the lives of students with disabilities by examining, and often exclusively, only their views and/or support staff. However, this approach leaves out other parties such as lecturers, family members, administrators and management involved in the lives of students with disabilities, whose experiences and perceptions are important for the improvement of disability policy and practice.

Some studies report that lecturers lack disability awareness. In one such study, Crous (2004) found that 67% of students with disabilities believed that their lecturers had limited knowledge of disability. Where lecturers thus seemed unhelpful, for example, in terms of time allocated to complete assignments, students often related it to their lack of awareness regarding disability, rather than their unwillingness to help them. The lack of awareness on the part of lecturers was also highlighted by Mayat and Amosun (2011) in their study, which explored the perceptions of academic staff of admission of students with disabilities, and their accommodation once accepted into a Civil Engineering programme at a South African university. Mayat and Amosun (2011) observed that students with disabilities in South Africa are still excluded from certain academic fields like Engineering and Natural Sciences. Even though the five participating staff members expressed willingness to teach students with disabilities, they showed some reservations. The authors argue that staff members were concerned about the perceived limitations of students with disabilities. They expressed concern that students with disabilities would not be able to meet all the course requirements. One lecturer even wondered whether students with disabilities would not be an ‘embarrassment’ to their able-bodied peers (Mayat & Amosun 2011:55). Although these unjustified perceptions will likely vary depending on the type and severity of impairment, the issues raised from these two studies makes a case for continued probing from the lecturers’ side on how they perceive disability matters at universities and work on possible avenues towards full academic inclusion and participation of students with disabilities.

Understanding lecturers’ views regarding disability at universities is important as the behaviour of some lecturers exclude students with disabilities. This was highlighted in a study by van Jaarsveldt and Ndeya-Ndereya (2015) on the e-learning needs of students with disabilities at a South African university. Lecturers’ responses in this study indicated that while some lecturers used their personal agency to respond to the needs of students with disabilities, some lecturers distanced themselves from the responsibility of providing support to students with disabilities. Those who distanced themselves displayed a lack of involvement with the students and tended to refer them to the Disability Unit (DU) at the institution. Van Jaarsveldt and Ndeya-Ndereya (2015) then argue that although higher education institutions’ disability policies are necessary, personal responsibility from lecturers is also essential in bringing about inclusive campuses.

Some students perceive that lecturers’ lack of disability awareness results in them failing to make necessary provisions (Matshedisho 2010). Swart and Greyling (2011) found that students in the Humanities and Social Sciences were more positive about the support they receive from lecturers than other students in the Natural, Economic and Business Sciences. Focusing on one higher education institution, Ohajunwa et al. (2014) investigated whether, and how, disability issues are included in the teaching and research of three faculties: Health Sciences, Humanities, and Engineering and the Built Environment at the University of Cape Town. Similar to Swart and Greyling (2011), this study reveals low levels of disability inclusion and disability not being viewed as an issue of social justice. However, there were pockets of inclusion, the nature of which differed from faculty to faculty, for example, out of 35 participants across the three faculties, 31 indicated that they include disability issues in their teaching (Ohajunwa et al. 2014:108). They went on to report that in the Faculty of Engineering and the Built Environment, disability was included as an issue of legislation, space and environment. At the Faculty of Humanities the focus was on the socio-cultural and economic impact of disability. The Faculty of Health Sciences introduced disability with an emphasis on individual impairment, environmental effects, community-based rehabilitation and inclusive development, as well as the prevention and management of disability. The authors rightly proposed the creation of an institutional system that will build the capacity of lecturers to include disability in teaching and research across faculties, in line with the university’s transformation agenda. The fragmentation of how universities through their departments respond to disability, as shown in this study, calls for an urgent need to understand how different universities are addressing the needs of students with disabilities.

These studies clearly show how lecturers from different departments and universities understand and view academic lives of students with disabilities. Lecturers are often the first point of contact for students, especially in the first year of study (Bierwert 2002). Research cited above indicated that the learning attitudes and approaches of lecturers are likely to have an impact on students’ learning (Cameron & Nunkoosing 2012). These assertions made the exploration of lecturers’ experiences and perspectives justified at the universities in this study, as no such study as this has been undertaken before in South Africa. We considered that the lecturers would provide insight given studies already done at other universities. This would further inform the debate about the inclusion of students with disabilities in higher education.

## Methodology

Purposive sampling was employed to recruit participants into a qualitative study. Participants included 14 students with various types of impairments (hearing, physical, visual and mobility), 4 able-bodied lecturers and 3 disabled DU staff. This paper only reports the data from lecturers. They were recruited through their respective Heads of Departments. A hard copy information sheet was provided to every lecturer. This was accompanied by a conversation clarifying the objectives of the study before they signed the consent form. Data were collected through in-depth interviews. Findings from this paper are based on the narratives of four lecturers, two from University of the Free State (UFS) and two from University of Venda (UniVen), about their experiences with students with disabilities in higher education, how their socio-cultural backgrounds influence their perceptions regarding disability and their role in their university lives. Their names have been anonymised. Each interview lasted between 40 and 60 minutes and data were digitally recorded and transcribed. The transcribed interviews were then analysed with the help of NVivo software by coding themes to generate tentative descriptive labels. Although this sample is not large enough to make generalisations, future studies that utilise mixed research methods might generate generalisable data. Bassey (1981) makes a valuable point by stating that the *relatability* of a case study is as equally important as *generalisability*. In his opinion, an important criterion for judging the merit of a study is the extent to which data are sufficient and appropriate for someone working in a similar situation or condition to make policy decisions based on what is described in the study. It is our hope that this paper, with data from a sample of four lecturers, is valuable for inclusive policy and from which further studies can be developed. To set the scene and give this discussion a context, we provide the lecturer profiles in Table 1.

**TABLE 1:** Profile of lecturers.

Name	Institution	Race	Gender	Number of years in practice	Field
Prof J	UFS	White	Male	More than 25 years	Education
Dr H	UFS	White Male	Less than 10 years	Law
Prof M	UniVen	Black	Male	More than 15 years	Human and Social Sciences
Mr L	UniVen	Black	Male	Less than 5 years	Mathematical and Natural Sciences

UFS, University of the Free State; UniVen, University of Venda.

## Findings

Findings are organised into four themes: lectures’ attitudes towards students with disabilities; disability awareness training; institutional disability arrangements and the preparedness of students with disabilities for higher education.

### Attitude of lecturers towards students with disabilities

Both negative and positive attitudes towards students with disabilities were found. Below are some of the elements of negativity:

‘The only time that the faculty can know that a student has a disability is when we are informed about that. We cannot do anything if we don’t know that a certain student has a disability. I have been the teaching and learning manager within the faculty since last year but I have not seen any student coming to me saying that he/she has a disability and that he or she needs assistance…I think that disability issues should be dealt at the institutional level and not individually by each faculty or lecturer because it’s an issue that needs to be addressed at institutional level. Something like that should come from the institutional policies.’ (Dr H, male, lecturer)

‘How do I know that a student has a learning disability? If I just think of spellings, conceptualising and formulations, it’s a massive problem for most of our students.’ (Prof. J, male, lecturer)

These two lecturers are raising pertinent challenging issues faced by lecturers. However, underlying the above statements are features of shifting the blame from individual teaching staff to either the students with disabilities and/or their respective institutions. These lecturers’ views suggest a lack of understanding of diversity which leads to exclusion of students with disabilities in teaching and learning activities, and consequently to their failure at universities. While one can argue that the lecturers are referring to students with severe learning disabilities, this cannot be a justification for failing to attend to their needs. The fact that they would have succeeded in the pre-university education is a testimony that they have the potential of succeeding at the university. Challenges faced by students with disabilities are individualised in the absence of an integrated approach which takes into account individual factors as well as other external factors. This, unfortunately, leads to lecturers failing to make necessary provisions for students with disabilities. Dr H points to the fact that if the affected students do not disclose their disabilities, they cannot be offered help by the teaching and learning staff. Prof J thinks that learning disability is difficult to detect as some of the symptoms are related to challenges that are also faced by non-disabled students. It might be true that in an environment like the South African education system which still grapples with the effects of the apartheid system, distinguishing students facing learning challenges as a result of disabilities from those having challenges as a result of an unfair pre-university background is difficult. However, it cannot be a justification not to respond to the needs of students with disabilities. It is also important for lecturers to make some effort to understand why students with disabilities do not disclose their status and to come with measures that distinguish challenges faced by students with learning disabilities and those faced by non-disabled students in class.

Some university teaching and learning practices that are not related to disability but which affect how they transmit knowledge to all the students, including students with disabilities, were also mentioned at both universities. Large classes and limited resources were highlighted:

‘Some lecturers do not want to spend much time on one or two students because of pressure and demands coming from huge classes. In some classes there are over 500 students. It becomes tough for one lecturer to provide individual attention.’ (Prof. J, male, lecturer)

‘We only have two laboratory technicians who are supposed to help between 20 and 50 students daily. How can we work well under these conditions?’ (Mr L, male, lecturer)

This evidence from these lecturers shows areas of commonality regarding the challenges faced by students with disabilities and those faced by non-disabled students. These findings are important in challenging the idea of treating students with disabilities as a homogenous category as this overlooks the varied experiences among students.

On a positive note, not everything about lecturers’ responses to the needs of students with disabilities is negative. Some positive attitudes towards students with disabilities were reported and these resulted in positive outcomes for students with disabilities:

‘Some of our practical exercises in class cannot be taken by other students e.g. partially sighted students because some of the instruments we use. An endoscope e.g. has too much light inside which is not good for the eyes. We also use laser which again is not good for the eyes and the vernier callipers which are very sharp. In instances like these we make alternative practical exercises for the partially sighted students. The reason for these adjustments is that we want fair assessment for everyone.’ (Mr L, male, lecturer)

‘Assessments should be varied according to the barriers a student is experiencing. We try to be sensitive by having alternative assessments.’ (Prof. J, male, lecturer)

It is refreshing to note that lecturers appreciate alternative teaching, learning and assessment methods that cater for the needs of the students. However, these are ad hoc individual initiatives which leave students with disabilities at the mercy of their individual lecturers. It is, therefore, important for institutions to be clear in their policy documents on how all lecturers are supposed to provide alternative teaching, learning and assessments for students with disabilities.

### Disability awareness training

The lecturers report a lack of professional training in dealing with diversity matters, and particularly disability issues. This contributes to the lack of awareness, and ultimately to their ignorance and negative attitude towards disability issues:

‘The issue is that as lecturers, we are not trained to handle [*disability*] matters e.g. we have to deal with the slowness [*of some disabled students*] while at the same time you have big classes and you are rushing to meet department and faculty deadlines.’ (Prof J, male, lecturer)

‘I am a Physics lecturer and all I want is my students to get the fundamentals of Physics. I don’t think I am equipped to deal with disability matters.’ (Mr L, male, lecturer)

Another striking finding from this study is the acknowledgement by the lecturers of their lack of awareness on how to react and act when confronted by students with disabilities or disability issues in their practice:

‘How do I know that a student has a learning disability? If I just think of spellings, conceptualising and formulations, it’s a massive problem for most of our students.’ (Prof. J, male, lecturer)

However, while there is an acknowledgement of not knowing how to respond to disability challenges by these lecturers, some of their statements point to the existence of subtle negative attitudes:

‘It’s a punishment. I have to change the font size in a lecture with visually challenged students, a lecture which is supposed to be one hour takes me two hours for those guys.’ (Mr L, male, lecturer)

Mr L views his responsibilities as burdening. He seems not to view it as part of his job to make sure that all his students access teaching and learning in an equitable manner. While Mr L might be trying to portray the challenges of heavy teaching load placed on the lecturers and lack of appreciation regarding the academic needs of students with disabilities, disability awareness workshops emphasising the need to attend to academic needs of students with disabilities might be helpful for the lecturers.

Institutional arrangements also negatively affect lecturers in attending to the needs of students with disabilities:

‘Some buildings were built years ago without disabled students in mind. What can I do when I have classes in those buildings? Students in wheelchairs are entirely excluded.’ (Prof. M, male, lecturer)

In case of physical buildings and other institutional arrangements, lecturers might have less influence to bring about positive change. However, together with other stakeholders like students with disabilities, university management and government and private sector players, alternative arrangements and solutions might be found. This points to the fact that although lecturers in their individual capacities can act to bring inclusion and access for students with disabilities at universities, full inclusion for the success of students with disabilities is possible when all the stakeholders are included and are working together.

### Institutional disability arrangements

At the time of our research, there was no formal disability policy at UFS, while at UniVen a one-page policy document was provided. This leads to different, inadequate and fragmented ways of responding to the needs of students with disabilities at these universities.

Lecturers highlight that the administration and the students have an important part to play in creating a good environment for students with disabilities in the university:

‘On the application forms students are asked to declare disability status. The administration captures the data but as the lecturers we never receive this information from them afterwards. The administration must tell us in advance about the specific students who need special attention.’ (Mr L, male, lecturer)

This is indicative of the fact that lecturers need information and support to build inclusive campuses. In order for lecturers to create inclusive environments, it is necessary for them to be aware of disability matters. The current situation might result in students with disabilities performing poorly in academics as a result of the lack of support from lecturers.

Although some lecturers are generally supportive of students with disabilities, they sometimes feel overwhelmed by requests for individualised support and are unsure how to balance maintaining academic standards and accommodating the needs of students with disabilities. However, this need not be an either–or situation as the needs of students with disabilities can be provided while academic standards are being kept. This finding is the same as reported by Riddell, Tinklin and Wilson (2005) who suggest that not having enough time to pay attention to each student is one of the reasons lecturers are reluctant to change or adapt their teaching methods. This links to an increasing issue of pressure of increased workload raised by academics in South African higher education system.

### Students with disabilities’ preparedness and their attitudes at universities

It emerged from the interviews with lecturers that some students with disabilities display negative attitudes and a lack of preparedness for higher education. Consequently, this affects their full inclusion in higher education. For example, some students with disabilities are exposed to new technology or ways of doing things, which are meant to help them, only after they have been admitted into university:

‘Some students come here not knowing e.g. how to use braille materials. It’s a mountain to climb.’ (Prof. M, male, lecturer)

Prof. M’s statement indicates the challenges faced by students with disabilities at universities in order for them to access teaching and learning. The same challenge of students with disabilities being exposed to different arrangements for the first time in the university was highlighted by Prof. J also who complained that as university lecturers, ‘we cannot make them [*students with disabilities*] recover all that has been lost at school…’. There are interventions that have been put in place at the two case study universities to help students with disabilities. However, these interventions are discipline-focused, for example, having alternative practical exercises and assessment criteria in Science and Information Technology subjects. Existing interventions do not cover all aspects of students with disabilities’ lives and other departments do not have any interventions.

Besides a lack of preparedness, it is reported that some students with disabilities lack agency to take initiative that might help them to flourish in higher education:

‘If a student with disabilities experiences a barrier but communicates well with a lecturer things are likely to run smoothly but the student must come to the fore. It is very tough if there are invisible disabilities that are not reported and it’s not known by the lecturers.’ (Prof. J, male, lecturer)

‘Some students with disabilities have negative attitude towards learning. I expect my students to be at a certain level of competence in my course at a certain time regardless of one’s status but if someone wants to be treated in a special way in school work because of a disability, it becomes a challenge and definitely people like that fail.’ (Mr L, male, lecturer)

Prof. J and Mr L highlight important aspects which need to be examined. Firstly, though they want students with disabilities to flourish, they seem not to be encouraging them by inviting them to discuss their needs. These lecturers seem to distance themselves from the responsibility of providing support to students with disabilities. Secondly, their expressions convey an ‘us versus them’ discourse (van Jaarsveldt & Ndeya-Ndereya 2015:207). This leads to poor academic performance among students with disabilities. As pointed out by Morris (2001), social inclusion cannot be accomplished as long as conditions which maintain exclusion stay untouched. As such, we need to pay attention to the everyday language and how people with disabilities are represented. Besides a lack of training on diversity matters, individual agency on the part of the lecturers to enhance their own understanding of disability is also vital. These will help foster disclosure of disability status among students with disabilities who fail to disclose because of stigma (De Cesarei 2015).

Responsibility lies with the entire university population. Moreover, concerned lecturers who are aware of, and take an interest in, students with disability issues make an effort to learn about disabilities. Greyling’s (2008) claim is valuable. She says that although DUs or divisions for student support services are crucial in providing individual support and addressing institutional barriers, they should not be seen as the exclusive providers of support to students with disabilities. Not only are the universities supposed to remain responsible for the transformation of different departments, but all relevant players are responsible for creating an inclusive environment.

## Ethical consideration

Ethical clearance was given at the two institutions. At the University of the Free State, the reference number is UFS-HUM-2014-46.

## Discussion

Lecturers’ narratives highlight that in most cases, they are aware of the need for creating an inclusive atmosphere for all students. However, they face challenges in their quest to promote and create barrier-free environments for students with disabilities. Some of these challenges are influenced by institutional policies and practices. This might be stemming from a concern or a belief that accommodating the needs of students with disabilities might lower academic integrity or is unfair to students who are not disabled (Fuller, Bradley & Healey 2004). While previous studies (Fuller et al. 2004; Matshedisho 2010; Moriña, Cortés & Melero 2014) portray students with disabilities as victims and lecturers as perpetrators of social injustices, these four lecturers highlight that teaching staff are also victims of a system that fails to equip them to deal with diversity challenges in higher education. They also point to the fact that in some instances, students with disabilities create barriers to learning by their attitudes towards learning. Thus, disability policies need to be multi-focused – targeting students with disabilities as well as encouraging them to be an active agency in their own lives.

With regard to teaching staff’s awareness of disability issues and their attitudes towards students with disabilities, data show that positive attitudes towards disability depend on the initiatives by the individual lecturers. This is not surprising considering how lecturers are appointed and promoted in these universities. In most cases, a lecturer is appointed into his or her field of expertise based on the academic record for the related courses and the ability to conduct research in his or her field. Except for some programmes (e.g. Education where subjects like classroom management, pedagogies and curriculum studies are taught), in other disciplines, this is left to the lecturer concerned to handle. This is contrary to the UFS’ mission of ‘Advancing social justice by creating multiple opportunities for disadvantaged students to access the university’ (UFS 2016), or UniVen’s mission statement, ‘responsive to the development needs of the Southern African region, using appropriate learning methodologies and research’ (UniVen 2016). In practice, the advancement of social justice and appropriate learning methods for all students are missing at these two universities. Increased expectations on lecturers such as teaching large classes with around 700 students (*ibid*.) make it difficult to dedicate time to the needs of all students.

Lecturers highlighted another dilemma which confronts university teaching staff, that is, the need to balance classroom management and the need to reach the required departmental mandates, for example, taught modules delivered in a given timeframe. In some cases, this challenge is acknowledged and corrective measures are put in place. University staff are, among other performance measures, evaluated by the amount of hours spent delivering lectures to students. As such, the need to attend to pedagogical issues (e.g. paying individual attention to the needs of students with disabilities) is relegated as a secondary issue.

The lack of a sound relationship between lecturers and students with disabilities has a negative effect on the inclusion and participation of students with disabilities. Ginsberg and Wlodkowski (2009) express this:

…teaching methods and educational environments that motivationally favour particular learners to the exclusion of others are unfair and diminish the chances of success for those learners discounted or denied in this situation. (p. 32)

It is, therefore, important to pay attention to these issues in an attempt to create inclusive environments. Stojanovska-Dzingovska and Bilic (2012) report in their study that lecturers kept a distant from students with disabilities intentionally as they were afraid of offending their students by using inappropriate idiomatic expressions. Furthermore, Swart and Pettipher (2011) argue that beliefs and attitudes are directly translated into actions and educational practices, and inform decision-making. They further state that attitudes about diversity can either be a barrier to or an enabler in the realisation of an inclusive environment. While participating lecturers did not show strong evidence of reflection behaviour, lecturers need to be aware and be reflective of their perspectives and behaviour. Self-reflection, which involves deep inward looking into every action is critical for lecturers to become more aware and active in meeting the needs of all students. This is only possible if lecturers are willing to self-examine their own conceptions. However, short awareness seminars can help in this regard.

Lecturers in this study had varied backgrounds (race, institutional affiliation, fields of teaching and number of years in the teaching profession). Although this was not the focus of this study, one cannot produce any link between lecturers’ biographic characteristics and their perceptions and attitudes regarding disability and students with disabilities in higher education. This differs from previous studies (Rao 2002; Rao & Gartin 2003; Vogel et al. 1999), which found a positive relationship between discipline (education, humanities and architecture), age (junior v senior lecturers) and experience with teaching students with disabilities, with the willingness to provide accommodations and support to students with disabilities. A possible explanation for this might be the limited number of lecturers involved in this study. As highlighted in this and other studies (Hadjikakou & Hartas 2008) that lecturers are not trained to deal with disability issues, it is important for lecturers to receive professional development through disability awareness workshops, emphasising, for example, different types of impairments, the importance of an inclusive environment, how to encourage disclosure among students with disabilities, how to teach and assess the progress of students with disabilities without excluding anyone. Attending to identity, stigma, self-worth and self-awareness issues in these workshops will also be important.

## Conclusion

This paper examined the role, perceptions and experiences of lecturers regarding disability issues at two South African universities. In trying to create inclusive campuses, lecturers face challenges emanating from both internal and external factors. External factors include absence of a national disability policy for higher education in general and for universities, in particular. Internal factors include the lack of knowledge, responsibility and skills in addressing the needs of students with disabilities. Although different policy statements refer to the rights of students with disabilities in South Africa, they do not completely spell out how to implement the imperatives raised. This lack of guiding frameworks results in universities approaching disability differently, resulting in ad hoc and uncoordinated efforts towards disability matters. While institutional policy frameworks are important, personal responsibility on the part of the lecturers in expanding the opportunities of all students is important. Self-reflective curriculum is vital in this case for the creation of a student-centred approach that enhances learning for all students. Collaborative efforts among all the stakeholders (academic staff, supporting staff, administration and students with disabilities) are required to create a supportive education system and make inclusion of students with disabilities in higher education a reality.

Inclusion agenda can succeed if interventions account for and address dominant barriers that lecturers face in their quest to create inclusive environments. More needs to be done to help lecturers in South Africa to appreciate and deal with diversity issues, especially disability. There needs to be a shared sense of responsibility among all lecturers and other university stakeholders for meeting the needs of students with disabilities.

## Footnotes

1 In this paper, disability refers to disadvantages caused by multiple factors (social, economic, political, environmental and personal) on people with various impairments.

2 These include workers, mature students, female students and disabled students.

3 This paper is entitled ‘Special Needs Education: Building an inclusive education and training system’.
